# Long noncoding RNA ACART knockdown decreases 3T3-L1 preadipocyte proliferation and differentiation

**DOI:** 10.1515/biol-2022-0552

**Published:** 2023-02-09

**Authors:** Renyan Huang, Chenyan Shi, Guobin Liu

**Affiliations:** Vascular Surgery Department, Shuguang Hospital Affiliated to Shanghai University of Traditional Chinese Medicine, Shanghai, China; Department of Otolaryngology, Shuguang Hospital Affiliated to Shanghai University of Traditional Chinese Medicine, Shanghai, China

**Keywords:** lncRNA, *Acart*, adipogenesis, PPARγ, C/EBPα

## Abstract

Obesity is a main risk factor for diabetes and cardiovascular disorders and is closely linked to preadipocyte differentiation or adipogenesis. Peroxisome proliferator-activated receptor γ (PPARγ) is an indispensable transcription factor in adipogenesis. A newly identified long noncoding RNA, *Acart*, exerts a protective effect against cardiomyocyte injury by transactivating PPARγ signaling. However, the function of *Acart* in preadipocyte differentiation is unclear. To investigate the function of *Acart* in adipogenesis, a well-established preadipocyte, the 3T3-L1 cell line, was induced to differentiate, and *Acart* level was assessed during differentiation using quantitative real-time PCR. The biological role of *Acart* in adipogenesis was analyzed by assessing lipid droplet accumulation, PPARγ and CCAAT/enhancer-binding protein α (C/EBPα) expression, and 3T3-L1 cell proliferation and apoptosis after *Acart* silencing. We found that *Acart* level was promptly increased during preadipocyte differentiation *in vitro*. *Acart* was also significantly upregulated in obese mouse-derived subcutaneous, perirenal, and epididymal fat tissues compared with nonobese mouse-derived adipose tissues. Functionally, *Acart* depletion inhibited preadipocyte differentiation, as evidenced by a significant decrease in lipid accumulation and PPARγ and C/EBPα expression levels. *Acart* silencing also inhibited 3T3-L1 cell proliferation, whereas *Acart* overexpression accelerated 3T3-L1 cell proliferation and decreased cell apoptosis. Taken together, the current results reveal a novel function of *Acart* in regulating preadipocyte proliferation and differentiation.

## 1 Introduction

Epidemiological studies show that obesity has become a public health challenge worldwide, as there are approximately 2 billion obese people [1,2]. Obesity is closely correlated with an increased risk in multiple serious disorders, including diabetes, cardiovascular disorders, and cancers [3,4], and is closely associated with preadipocyte differentiation or adipogenesis [5]. Adipogenesis is pivotal to adipose tissue mass accumulation and obesity occurrence [6,7]. Although adipogenesis is a complex process involving many proteins and noncoding RNAs (ncRNAs), it is well known that peroxisome proliferator-activated receptor γ (PPARγ) and C/EBPα are two critical factors in adipogenesis [8,9].

Long noncoding RNAs (lncRNAs) are large and diverse noncoding transcripts that are >200 nucleotides in length and do not have protein-coding potential [10]. Over the past decade, lncRNA has attracted widespread attention from all fields of biology, such as chromatin remodeling, cell differentiation, cancer biology, and metabolism [11,12]. With the advancements of next-generation sequencing and microarrays, great progress has been made in identifying differentially expressed lncRNAs during adipogenesis. The results from RNA-sequencing analysis revealed that 175 lncRNAs are significantly dysregulated more than 2-fold during adipocyte differentiation [13]. Zhao et al. identified 21 lncRNAs enriched in brown adipose tissue that are upregulated during brown adipocyte differentiation through a microarray analysis that covered 31,423 annotated lncRNAs [14]. They further demonstrated that the overexpression of lncRNA-*Blnc1* accelerates brown adipocyte differentiation. Lo et al. showed that 68 lncRNAs are dysregulated in high-fat diet (HFD)-fed obese mice and that lncRNA-*Leptin* accelerates adipogenesis by maintaining leptin expression [15].

Emerging studies have demonstrated that many lncRNAs control adipogenesis by regulating PPARγ signaling, which is a critical factor for adipogenesis. For instance, the overexpression of lncRNA-*Plnc1* accelerates preadipocyte differentiation by activating PPAR-γ signaling [16]. lncRNA-U90926 expression is reduced during preadipocyte differentiation. Forced expression of lncRNA-*U90926* suppresses adipogenesis by inhibiting PPAR-γ transactivation [9]. lncRNA-*Acart* (hereafter named *Acart*) is a newly identified lncRNA in fibrotic cardiac tissue that attenuates cardiomyocyte injury by activating PPARγ signaling [17]. Based on the above findings, the biological role of *Acart* in adipogenesis was investigated.

## 2 Materials and methods

### 2.1 Animal model and adipose tissues

Adult male C57BL/6 mice were purchased from the Shanghai Experimental Animal Center, housed at an optimal temperature and humidity, and given free access to water and food. The animal work was performed in accordance with the guidelines and regulations of the CCCA and the approval of the Animal Ethics Committee of Shanghai Shuguang Hospital (No. PZSHUTCM220124012). Obesity was achieved by feeding the mice an HFD (catalog number: D12492, Research Diets, NJ, USA) for 7 weeks (*n* = 5) [18]. Normal mice (*n* = 5) were fed a basal diet (BD, catalog number: D12450B, Research Diets). Mice were euthanized via isoflurane inhalation, and then, subcutaneous, perirenal, and epididymal fat was collected when the difference in body weight between the two groups was 30% [9].


**Ethical approval:** The research related to animal use has been complied with all the relevant national regulations and institutional policies for the care and use of animals and has been approved by Animal Ethics Committee of Shanghai Shuguang Hospital (No. PZSHUTCM220124012).

### 2.2 Cell culture

A murine preadipocyte cell line, 3T3-L1, was purchased from the Cell Bank of the Chinese Academy of Sciences (Shanghai, China). These cells were maintained in complete medium (Dulbecco’s modified eagle’s medium supplemented with 10% fetal bovine serum; Product number: SLM-243-B; Sigma-Aldrich, MO, USA) in an incubator with 5% CO_2_ at 37°C, as described previously [19]. Two days after complete fusion, 3T3-L1 cells were induced to differentiate (Day 0, D0) using a previously described standard cocktail regimen: 0.5 mM IBMX (Product number: 15879; Sigma-Aldrich), 10^−6^ M dexamethasone (Product number: D4902; Sigma-Aldrich), and 5 µg/mL insulin (Product number: 12643; Sigma-Aldrich) (designated as MDI) [20]. Two days after this induction, the medium was replaced with complete medium supplemented with insulin (5 µg/mL) for 48 h. Finally, the cells were maintained in complete medium without any inducer.

### 2.3 RNA interference (RNAi) and overexpression

Oligomers for sh-*Acart* (forward, GATCCCCAGAAUCCCACACGUCAATTGCAATTGACGTGTGGGATTCTGGTTTTTT; reverse, CTAGAAAAAACCAGAAUCCCACACGUCAATTgcAATTGACGTGTGGGATTCTGGG) or shNegative Control (sh-NC) were synthesized from Sangon Biotech (Shanghai, Chain) and cloned into a pGE1 vector. Oligomers for sh-NC were as follows: forward, GATCCTTCTCCGAACGTGTCACGTTTGCAAACGTGACACGTTCGGAGAATTTTTT; reverse, CTAGAAAAAATTCTCCGAACGTGTCACGTTTGCAAACGTGACACGTTCGGAGAAG. sh-*Acart* and sh-NC were synthesized and cloned into pLKO.1 vector (Product number: 1.8787; Addgene, USA) to construct recombinant plasmids pLKO.1-sh-*Acart* and pLKO.1-sh-NC, respectively. pLKO.1-sh-*Acart* and pLKO.1-sh-NC were transfected into 3T3-L1 cells using lipofectamine 3000 (Product number: L300001; Invitrogen, CA, USA), and the interference efficiency was validated using quantitative real-time PCR (qRT-PCR) after 48 h. The recombinant plasmid of pcDNA-*Acart* was constructed to overexpress *Acart* in 3T3-L1 cells.

### 2.4 Oil Red O staining

After treatment with the specified reagents, 3T3-L1 cells (2 × 10^5^ cells per well in six-well plates) were fixed with 4% PFA (Product number: P1110; Solarbio, Shanghai, China) and stained with Oil Red O (Product number: 08010-5; Solarbio) to assess lipid accumulation, as previously described [21]. After staining, cells were visualized and photographed using a microscope (Olympus, Tokyo, Japan).

### 2.5 qRT-PCR

Total RNA was extracted with TRIzol reagent (Product number: T9424; Sigma-Aldrich). Genomic DNA was degraded using DNase I (Product number: EN0521; Thermo Fisher Scientific) prior to reverse transcription. cDNA was synthesized using Moloney’s murine leukemia virus reverse transcriptase (Product number: M1302; Sigma-Aldrich) and random primers (3 µg/µL; Product number: 48190011; Invitrogen, CA, USA). qRT-PCR was performed in triplicate on a ABI 7500-Fast Real-Time PCR System (Applied Biosystem, CA, USA) using a OneStep qPCR kit (Product number: 210210; Qiagen, Duesseldorf, Germany). The temperature protocol was 95°C for 15 min, followed by 39 cycles of 95°C for 15 s and 58.5°C for 15 s. Beta (β)-actin served as an internal control. The mRNA levels were analyzed using the 2^(−ΔΔCT)^ method [22]. The primer sequences are shown in Table 1. The specificity of qRT-PCR product was ascertained through gel electrophoresis and melt curve analysis.

**Table 1:** qPCR primers used in the study

Gene	Sense (5′–3′)	Anti-sense (5′–3′)	*T*_m_ (°C)	PCR product size (bp)	Amplification efficiency (%)
ACART	TCAGTGGATTTATGTCTGTTGGG	AGAATGTACGTGTGTGTGTGTGTG	59.3/58.3	162	94.9
PPARγ	ACAGTTGATTTCTCCAGCATTTC	GCAGGTTCTACTTTGATCGCAC	58.0/59.3	134	95.3
C/EBPα	CGCAAGAGCCGAGATAAAGC	AGGCAGCTGGCGGAAGATG	60.4/63.6	150	92.7
FABP4	GGTGAAGAGCATCATAACCCTAG	ATAACACATTCCACCACCAGC	57.8/57.2	120	95.8
AdipoQ	CGACCAGTATCAGGAAAAGAATG	GGAAGAGAAGAAAGCCAGTAAAT	58.5/56.4	164	92.5
β-Actin	AATCGTGCGTGACATCAAAGAG	AGGAAGGCTGGAAAAGAGCC	60.3/60.1	176	96.2

Primer concentrations: 10 µM.

### 2.6 Western blot

3T3-L1 cells were lysed with RIPA buffer (Product number: R0010; Solarbio), and the protein concentration was quantified with a BCA protein assay kit (Product number: PC0020; Solarbio). For western blot analysis, equal amounts of protein were loaded on a 10% sodium dodecyl sulfate polyacrylamide gel electro-phoresis gel and then electrotransferred to polyvinylidene fluoride membranes (Product number: 1620177; Bio-Rad, CA, USA). The membranes were blocked with 5% nonfat milk for 90 min and then treated with antibodies against PPARγ (1:1,000, ab178866; Abcam, CA, USA), C/EBPα (1:800, ab40764; Abcam), BCL-2 (1:1,500, ab196495), BAX (1:3,000, ab32503), and β-actin (1:2,000, ab8226; Abcam) overnight at 4°C. An anti-rabbit secondary antibody (1:8,000, ab288151; Abcam) was used to treat the membranes, and an ECL kit (Pierce, IL, USA) was applied to visualize the membranes.

### 2.7 Cell proliferation

Cell proliferation assays were performed with cell counting kit (CCK)-8 reagent (Product number: CA1210; Solarbio). Briefly, 24 h after transfection with sh-*Acart* or sh-NC, 3T3-L1 cells were plated into 96-well plates (4 × 10^3^ cells per well) and incubated for 1, 2, 3, and 4 days. Ten microliters of CCK-8 was added to the wells for 60 min, and then, the absorbance was measured at 450 nm with a microplate reader (Bio-Rad, CA, USA).

### 2.8 Edu incorporation assay

Twenty-four hours after transfection with sh-*Acart* or sh-NC, 3T3-L1 cells (0.5 × 10^5^ cells per well in 24-well plates) were induced to differentiate for 24 h and labeled with EdU (Product number: CA1170; Solarbio) and Hoechst (Product number: CA1170; Solarbio) for 1 h. The fluorescence of EdU and Hoechst was observed using a BX53 fluorescence microscope (Olympus).

### 2.9 Data analysis

Data are shown as the mean ± standard deviation from three independent experiments. Statistical analysis was carried out using two-tailed Student’s *t*-test with SPSS 20.0 statistical software (IBM, NY, USA). *p* < 0.05 was considered statistically significant.

## 3 Results

### 3.1 *Acart* level was upregulated during preadipocyte differentiation

**Figure 1:**
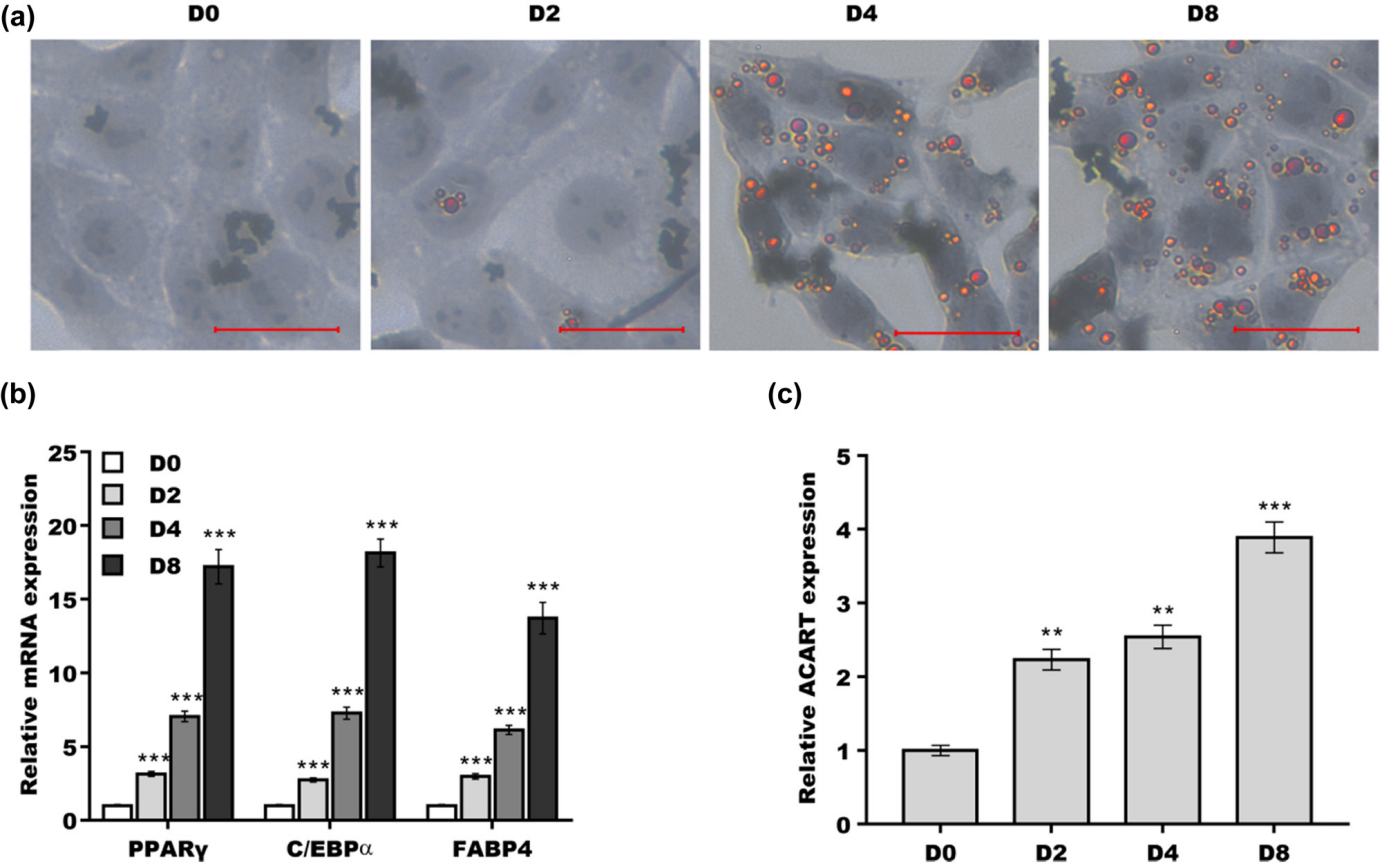
*Acart* level was increased during preadipocyte differentiation. (a) 3T3-L1 cells were treated with MDI differentiation medium, and lipid accumulation was assayed using oil red O staining at D0, D2, D4, and D8. Scan bar = 100 µm. (b) 3T3-L1 cells were treated with MDI, and then qRT-PCR analysis was carried out to assess the mRNA level of *Pparγ*, *C/ebpα*, and *Fabp4*. (c) 3T3-L1 cells were treated with MDI, and then, qRT-PCR analysis was carried out to assess *Acart* levels. **p < 0.01, ***p < 0.001.

**Figure A1:**
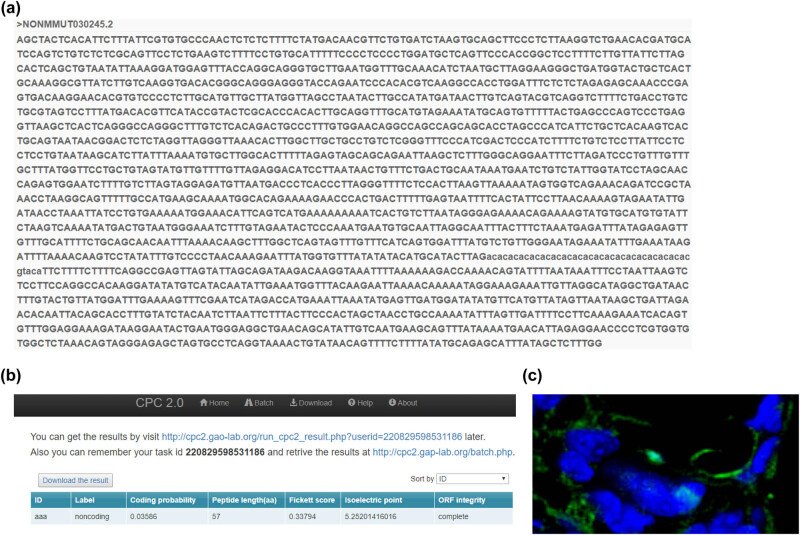
The basic information of *Acart*. (a) The full sequence of *Acart* (2193 bp). (b) The coding probability of *Acart* predicted by CPC2.0 tool (http://cpc2.gao-lab.org/). (c) The subcellular localization of *Acart* in 3T3-L1 cells.

*Acart* is an lncRNA with 2193 bp in length, mainly located in cytoplasm (Figure A1(a–c)). In a previous study, *Acart* was identified to play an important role in protecting against cardiomyocyte injury by activating PPARγ signaling [17]. Given that PPARγ is an essential transcription factor in adipogenesis, we first investigated whether *Acart* was dysregulated during preadipocyte differentiation. To this end, the 3T3-L1 cell line, a well-established preadipocyte, was treated with MDI differentiation medium to simulate adipogenesis *in vitro*. As shown in Figure 1a, MDI treatment significantly increased lipid droplet accumulation in a time-dependent manner. To further validate 3T3-L1 preadipocyte differentiation, the expression of major adipogenesis markers was assessed. The results from qRT-PCR analysis revealed that the expression of *Pparγ*, *C/ebpα*, and *Fabp4* exhibited a differentiation-dependent increase (Figure 1b). Based on these results, *Acart* level was assessed in 3T3-L1 cells during differentiation. Figure 1c shows that *Acart* levels were significantly upregulated during MDI-induced 3T3-L1 preadipocyte differentiation.

### 3.2 *Acart* was increased in adipose tissues from obese mice

**Figure 2:**
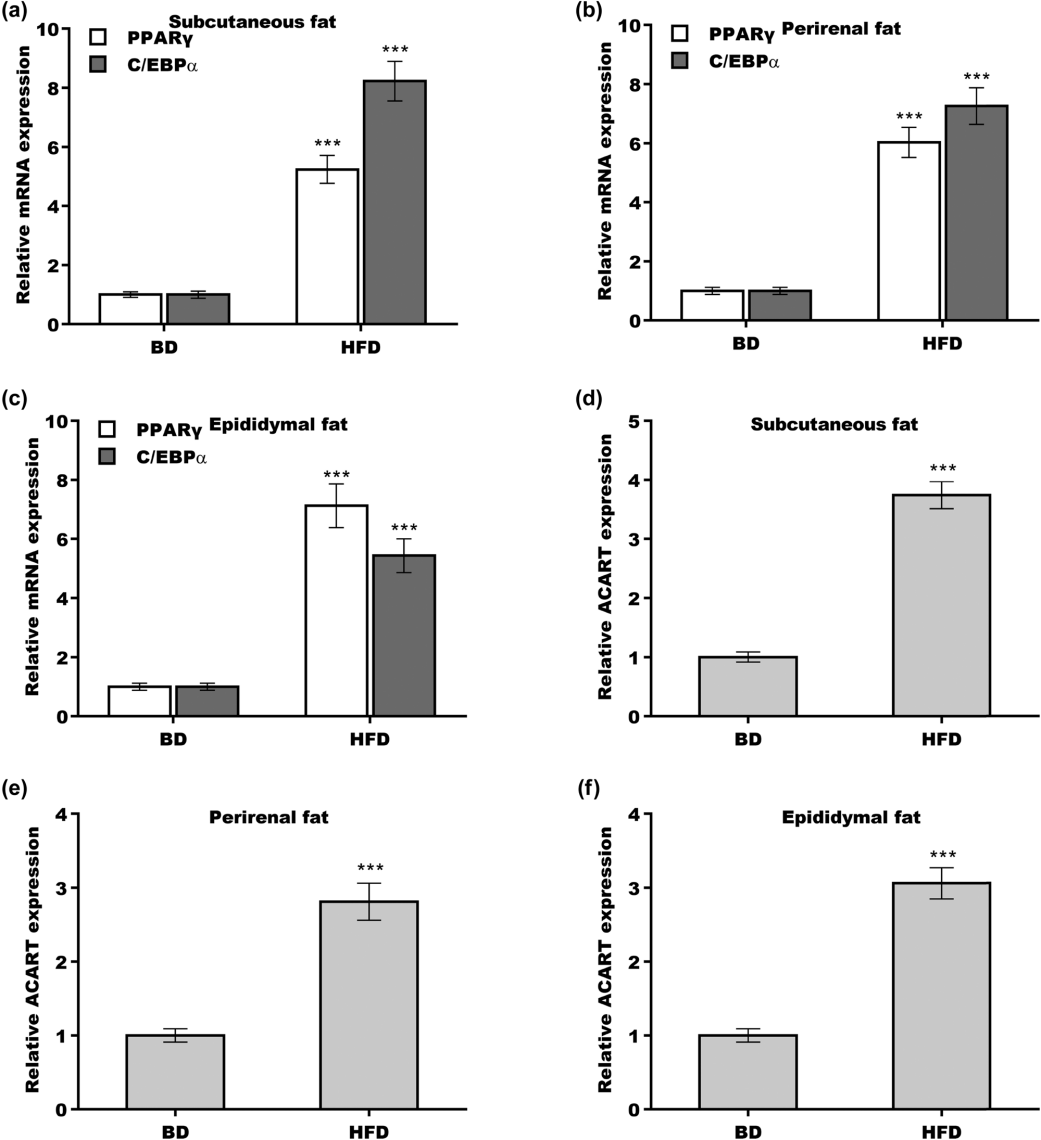
*Acart* level was increased in adipose tissues from obese mice. Subcutaneous (a), perirenal (b), and epididymal (c) adipose tissues were collected from HFD-fed mice and BD-fed mice, and *Pparγ* and *C/ebpα* mRNA levels were assessed using the qRT-PCR analysis. Subcutaneous (d), perirenal (e), and epididymal (f) adipose tissues were collected from HFD-fed mice and BD-fed mice, and *Acart* levels were assessed using the qRT-PCR analysis. ***p < 0.001.

**Figure A2:**
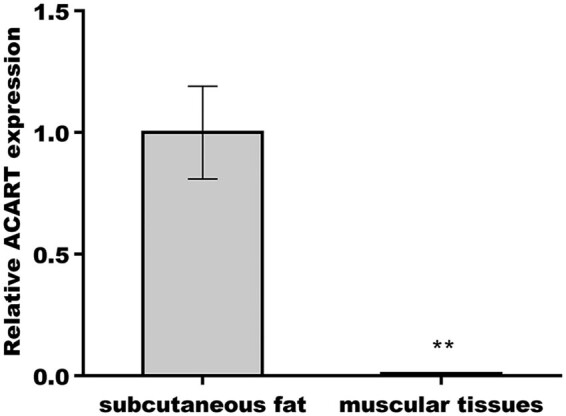
*Acart* expression in subcutaneous fat and muscular tissue. qRT-PCR analysis of *Acart* expression in subcutaneous fat and muscular tissue.

To investigate the association of *Acart* with obesity, subcutaneous, perirenal, and epididymal fat was obtained from HFD obese mice and BD normal mice. As expected, *Pparγ* and *C/ebpα* expression levels were higher in subcutaneous, perirenal, and epididymal adipose tissues from mice with HFD-induced obesity than from mice fed a BD (Figure 2a–c). More importantly, *Acart* level was also markedly increased in subcutaneous, perirenal, and epididymal adipose tissues in HFD-fed obese mice compared with BD-fed normal mice (Figure 2d and f). We did not detect *Acart* expression in muscular tissues (Figure A2).

### 3.3 *Acart* depletion inhibited preadipocyte differentiation

**Figure 3:**
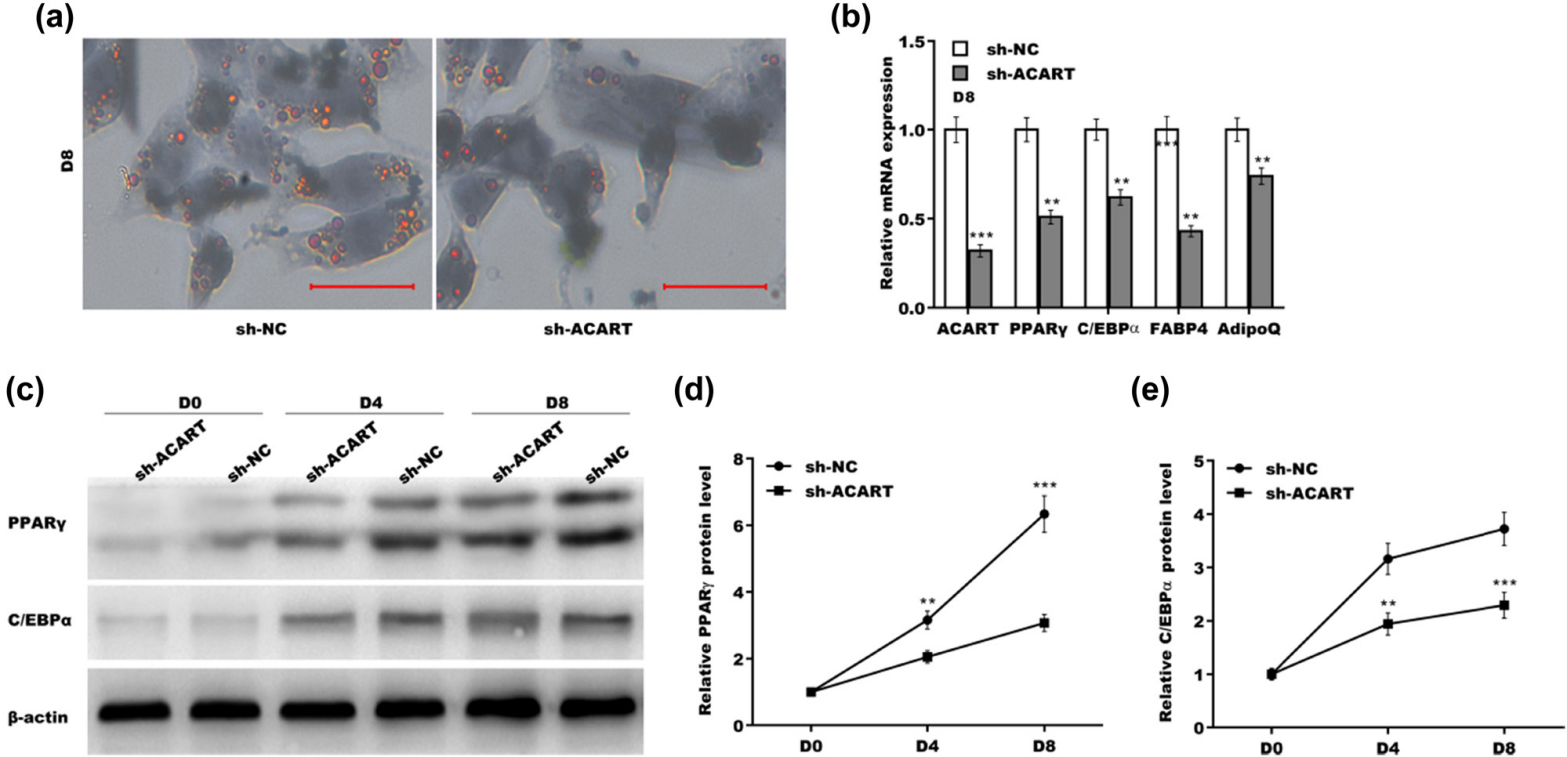
*Acart* depletion inhibited preadipocyte differentiation. (a) After transfection with pLKO.1-sh-*Acart* for 48 h, 3T3-L1 cells were treated with MDI for 8 days, and lipid accumulation was assayed using oil red O staining. Scan bar = 100 µm. (b) After transfection with pLKO.1-sh-*Acart* for 48 h, 3T3-L1 cells were treated with MDI for 8 days, and then qRT-PCR analysis was carried out to assess the mRNA levels of *Acart*, *Pparγ*, *C/ebpα*, *Fabp4*, and *Adipoq*. (c–e) After transfection with pLKO.1-sh-*Acart* for 48 h, 3T3-L1 cells were treated with MDI for 8 days, and then western blot (c) and quantitative (d and e) analyses were carried out to assess PPARγ and C/EBPα protein expression. **p < 0.01, ***p < 0.001.

To explore the biological role of *Acart* in preadipocyte differentiation, 3T3-L1 cells were treated with pLKO.1-sh-*Acart* to repress *Acart* level and then treated with MDI differentiation medium. The results from Oil Red O staining showed that *Acart* depletion resulted in a significant decrease in lipid droplet accumulation (Figure 3a). Figure 3b shows that the expression levels of four major adipogenesis markers (*Pparγ*, *C/ebpα*, *Fabp4*, and *Adipoq*) were decreased in *Acart*-deficient cells. The results from the western blot analysis revealed that PPARγ and C/EBPα protein expression levels were significantly decreased in *Acart*-deficient 3T3-L1 cells (Figure 3c–e).

### 3.4 *Acart* depletion inhibited preadipocyte proliferation

**Figure 4:**
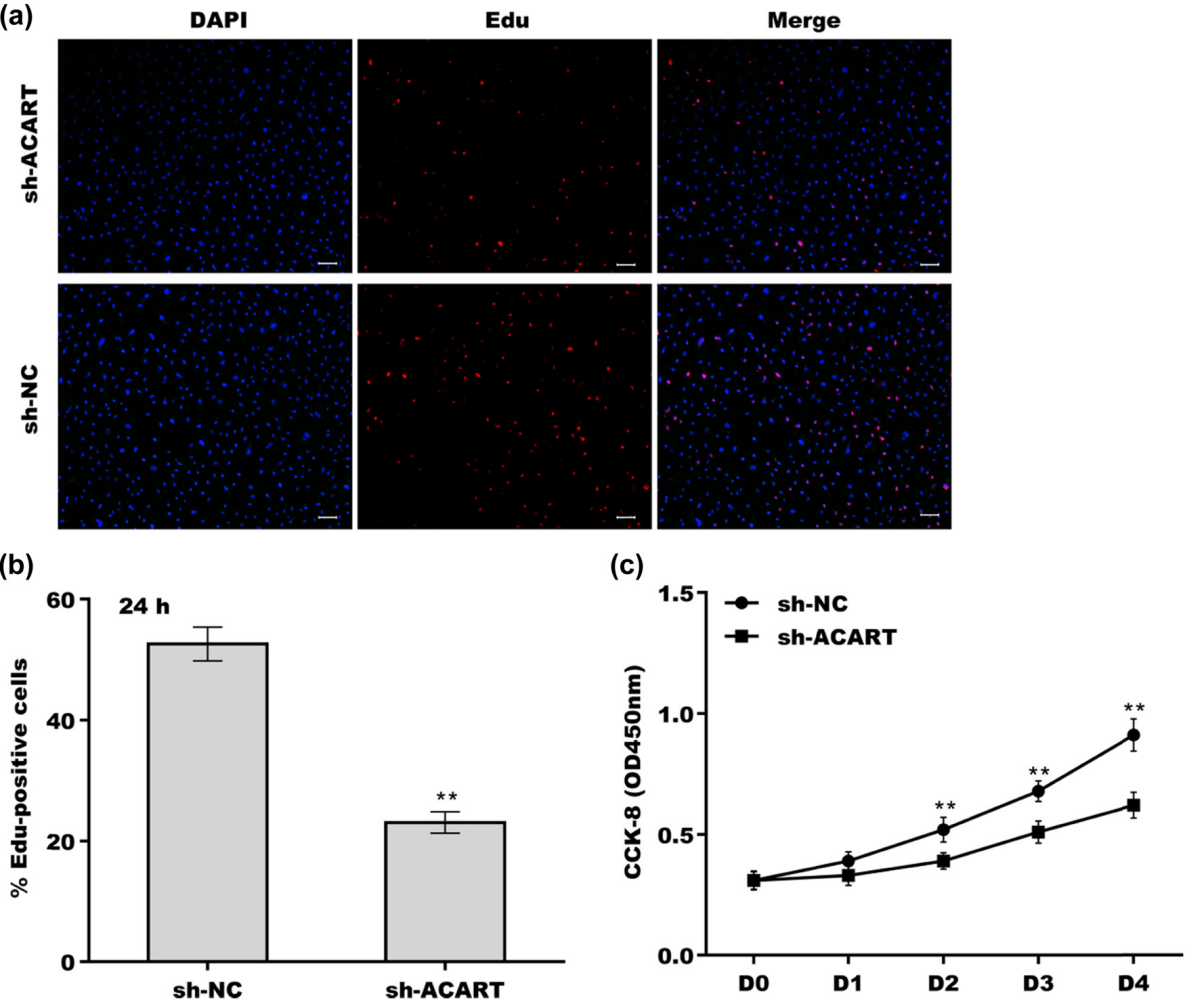
*Acart* depletion inhibited preadipocyte proliferation. (a and b) After transfection with pLKO.1-sh-*Acart* for 48 h, 3T3-L1 cells were treated with MDI for 24 h, and then, an EdU assay was carried out to assess cell proliferation. (c) The protein expression of BAX and BCL-2 was assessed in 3T3-L1 cells after *Acart* knockdown. (d) After transfection with pLKO.1-sh-*Acart* for 48 h, 3T3-L1 cells were treated with MDI for different time periods, and then, a CCK-8 assay was carried out to assess cell proliferation. **p < 0.01.

**Figure 5:**
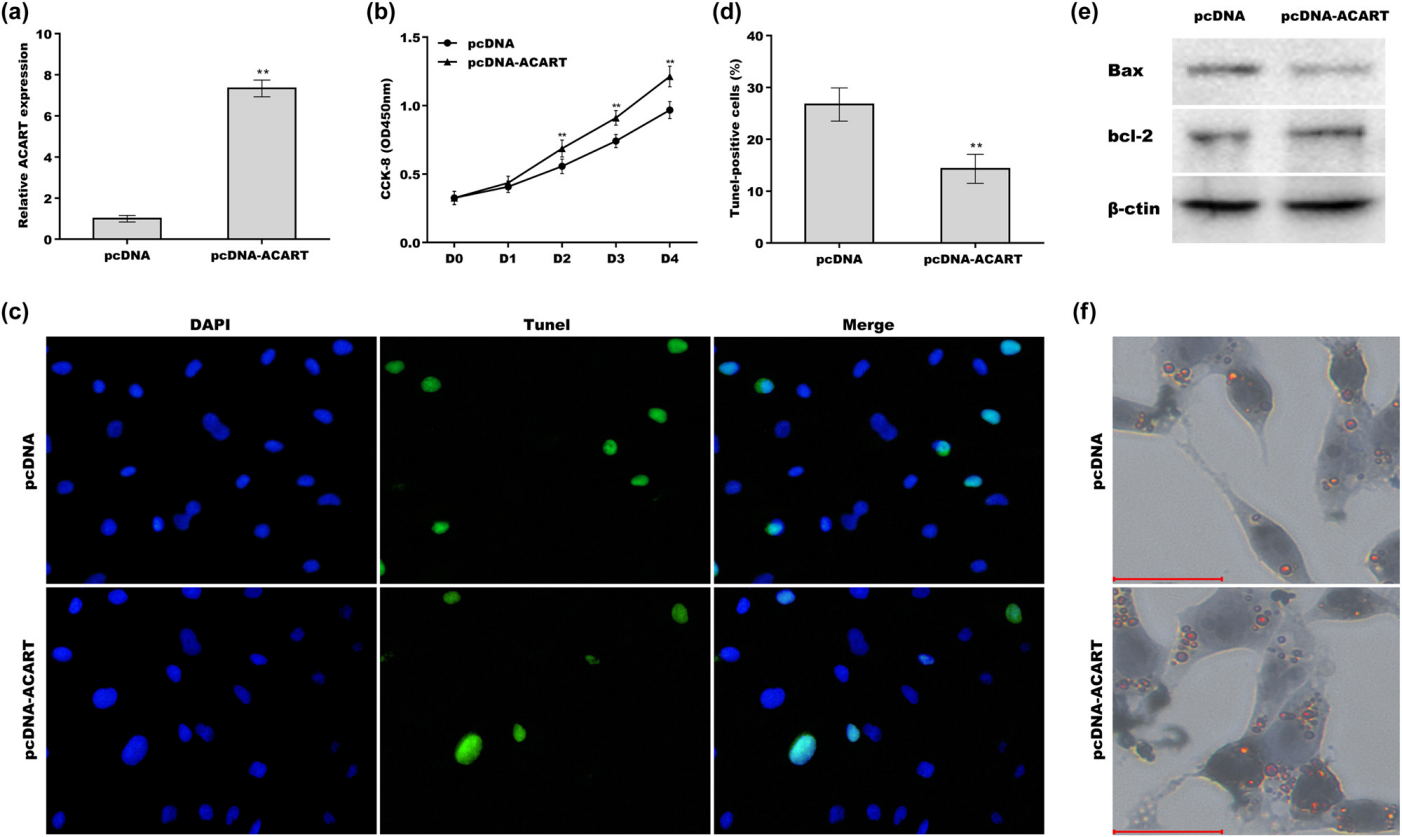
*Acart* overexpression accelerated preadipocyte proliferation and differentiation. (a and b) 3T3-L1 cells were treated with pcDNA-*Acart* and MDI for 24 h, and then, cell proliferation was assessed using the CCK-8 assay. After ACART overexpression, 3T3-L1 cells were treated with MDI for 24 h, and cell apoptosis (c and d) and lipid accumulation (f) were assayed using TUNEL and oil red O staining, respectively. (e) The protein expression of BAX and BCL-2 was assessed in 3T3-L1 cells after *Acart* overexpression. Scan bar = 100 µm. **p < 0.01.

Given that preadipocyte proliferation is an essential precondition for cell differentiation [19] and that *Acart* level is promptly increased during preadipocyte differentiation, the role of *Acart* in regulating preadipocyte proliferation was further explored. The results from the EdU assay showed that the EdU-positive cell proportion was significantly decreased in *Acart*-deficient cells compared with the negative control cells (Figure 4a and b). *Acart* knockdown increased BAX expression and decreased BCL-2 expression in 3T3-L1 cells, indicating that *Acart* knockdown facilitated cell apoptosis (Figure 4c). The CCK-8 assay revealed that cell proliferation was significantly repressed from day 2 to day 4 after *Acart* depletion (Figure 4d). Conversely, *Acart* overexpression accelerated preadipocyte proliferation (Figure 5a and b) and inhibited cell apoptosis (Figure 5c and d). *Acart* overexpression decreased BAX expression and increased BCL-2 expression in 3T3-L1 cells, indicating that *Acart* inhibited cell apoptosis (Figure 5e). The results from Oil Red O staining revealed that *Acart* overexpression resulted in a significant increase in lipid droplet accumulation (Figure 5f). These results demonstrate that *Acart* knockdown inhibits preadipocyte proliferation and differentiation.

## 4 Discussion

Adipocytes are a major component of adipose tissue and exert a critical role in homeostatically regulating whole body metabolism [23]. In addition to the primary function of controlling energy balance, adipocytes play an important role in regulating tissue metabolism [24], immune responses [25,26], insulin resistance [27], and cardiovascular disorders [23,28,29]. Preadipocyte differentiation is a complex multistep process involving many transcription factors and differentiation-related proteins and ncRNAs [8]. Here, we demonstrated that (1) *Acart* was upregulated during preadipocyte differentiation, (2) *Acart* was increased in adipose tissues from obese mice, (3) *Acart* silencing inhibited preadipocyte differentiation, and (4) *Acart* silencing inhibited preadipocyte proliferation. The current data identified the function of *Acart* in accelerating adipogenesis and indicated that *Acart* might exert an important role in adipogenesis.

PPARγ and C/EBPα synergistically control preadipocyte differentiation and are frequently used as markers to indicate adipose differentiation [30]. As a transcription factor, PPARγ can initiate the transcription of lipid metabolism-related mRNAs, including those for perilipin1 [31], fatty acid synthase [32], and insulin-like growth factor-binding protein-2 [33]. The mechanisms underlying PPARγ signaling activation are constantly being revealed. For instance, the upregulation of lncRNA-*Gm15290* in adipose tissue accelerates fat deposition by activating PPARγ [34].

Recently, transcriptomic analyses have identified thousands of abnormally expressed lncRNAs during adipogenesis, and functional analyses have revealed the biological role of lncRNAs in adipocyte development and differentiation. Through microarray analysis, Xiao et al. revealed that 677 lncRNAs are downregulated and 513 lncRNAs are upregulated in mesenteric white adipose tissues (mWATs) from fasted mice compared with the mWATs from control mice [20]. Functional studies further demonstrated that the downregulation of lncRNA-*Fr332443* contributes to adipogenesis by regulating RUNX1 and the mitogen-activated protein kinase pathway [20]. lncRNA-*Plnc1* could upregulate PPARγ2 transcription and expression by repressing the methylation of the PPARγ2 promoter and thus promote adipogenesis [16].

In a recent study, a newly identified lncRNA, *Acart*, exerted a protective effect against cardiomyocyte injury by transactivating PPARγ signaling. Given the key role of PPARγ in adipogenesis, we next investigated whether *Acart* regulates preadipocyte differentiation. As expected, *Acart* was increased during 3T3-L1 preadipocyte differentiation. Moreover, *Acart* was markedly increased in obese mouse-derived adipose tissues. Functionally, *Acart* knockdown significantly decreased 3T3-L1 preadipocyte proliferation and differentiation. There are major limitations of this study. First, the direct effect of *Acart* on regulating PPARγ signaling activation has not been validated in 3T3-L1 cells. Second, the underlying mechanism by which *Acart* accelerates 3T3-L1 cell proliferation and differentiation has not been identified. Generally speaking, lncRNAs enriched in the nucleus epigenetically control gene expression by regulating histone methyltransferase- or deacetylase-mediated chromatin remodeling [35,36]. lncRNAs located in the cytoplasm commonly function as competing endogenous RNAs to control target gene expression [37,38]. It is essential to identify the subcellular localization of *Acart* in 3T3-L1 cells to explore the underlying mechanisms.

## 5 Conclusion

*Acart* knockdown contributes to inhibit 3T3-L1 preadipocyte proliferation and differentiation.
